# INERTIAL LOAD SPRINT TRAINING IMPROVES NEUROMUSCULAR POWER IN OLDER ADULTS

**DOI:** 10.1093/geroni/igz038.608

**Published:** 2019-11-08

**Authors:** Jakob R Allen, Remzi Satiroglu, Emre Vardarli, Anthony Laico, Anthony Wolfe, Edward Coyle

**Affiliations:** 1 University Of Texas at Austin, Austin, Texas, United States; 2 University of Texas Medical Branch at Galveston, Galveston, Texas, United States

## Abstract

PURPOSE: Maximal strength and neuromuscular power decline after the fourth decade of life. The physiological cause of this progression is partly a selective atrophy of Type II or “Fast-twitch” muscle fibers. The aim of this study was to determine the efficacy of a novel, time-efficient form of exercise training involving repeated sprints lasting 4 s on an inertial load cycle ergometer, to promote increased neuromuscular power in males and females aged 50-70y. METHODS: Three days a week, forty older adults performed 15, 20, and 30 sprints per day over weeks 1, 2-4 and 5-8 of the study respectively. Rest intervals were progressively reduced from 56s, 41s, and 26s over the same time periods. Subjects began each sprint while stationary and then were instructed to pedal as hard and as fast as possible for 4 s. Maximal power was reached after 1-4 s of sprinting and measured after a familiarization day (PRE), and then post-training (POST). RESULTS: The average increase in maximal power was 10.5±1.4% from PRE to POST (616±41 to 684±48 watts) (p<0.01). CONCLUSION: Only ~2 min of cycle sprinting per training session was able to increase maximal neuromuscular power, an important physiological component of tasks of daily living throughout the lifespan.

